# Ugonin J Inhibits EMT and Migration in Prostate Cancer by Suppressing ADAM9 Expression

**DOI:** 10.32604/or.2025.074202

**Published:** 2026-02-24

**Authors:** Jo-Yu Lin, Tien-Huang Lin, Ya-Jing Jiang, Liang-Wei Lin, Kuan-Ying Lai, Yi-Chin Fong, Chih-Chuang Liaw, Chih-Hsin Tang

**Affiliations:** 1 Graduate Institute of Biomedical Sciences, China Medical University, Taichung, 404333, Taiwan; 2 School of Post-Baccalaureate Chinese Medicine, Tzu Chi University, Hualien, 970374, Taiwan; 3 Department of Urology, Buddhist Tzu Chi General Hospital Taichung Branch, Taichung, 427213, Taiwan; 4 Department of Pharmacology, School of Medicine, China Medical University, Taichung, 404333, Taiwan; 5 Department of Marine Biotechnology and Resources, National Sun Yat-Sen University, Kaohsiung, 804201, Taiwan; 6 Department of Sports Medicine, College of Health Care, China Medical University, Taichung, 404333, Taiwan; 7 Department of Orthopedic Surgery, China Medical University Hospital, Taichung, 404333, Taiwan; 8 Department of Orthopedic Surgery, China Medical University Beigang Hospital, Yunlin, 651012, Taiwan; 9 Graduate Institute of Natural Products, Kaohsiung Medical University, Kaohsiung, 807378, Taiwan; 10 Department of Medical Laboratory Science and Biotechnology, Asia University, Taichung, 413305, Taiwan; 11 Chinese Medicine Research Center, China Medical University, Taichung, 404333, Taiwan

**Keywords:** Prostate cancer, Ugonin J, epithelial–mesenchymal transition (EMT), a disintegrin and metalloproteinase domain-containing protein 9 (ADAM9)

## Abstract

**Background:**

Prostate cancer (PCa) is the most prevalent malignancy in men and often correlates with distant metastasis in its advanced stages. The study aimed to investigate the effects of Ugonin J, a natural compound isolated from *Helminthostachys zeylanica*, on PCa metastasis.

**Methods:**

The effects of Ugonin J on cell motility were assessed using migration and invasion assays. Reverse Transcription Quantitative PCR (RT-qPCR) and Western blotting were used to evaluate the impact of Ugonin J on mRNA and protein expression. RNA sequencing (RNA-seq) analysis was performed to investigate candidate mechanisms. Differential gene expression analysis in PCa patients was conducted using multiple databases.

**Results:**

Here, we reveal that Ugonin J blocks migration and invasion in PCa cells without affecting cell viability. RNA-seq analysis suggests that epithelial–mesenchymal transition (EMT) is potentially involved in Ugonin J’s anti-motility effects. Ugonin J also suppresses the expression of mesenchymal markers N-cadherin, β-catenin, Snail, and Slug while upregulating the expression of the epithelial marker E-cadherin. Furthermore, among 13 A disintegrin and metalloproteinase (ADAM) proteins, A disintegrin and metalloproteinase domain-containing protein 9 (ADAM9) is the most downregulated following Ugonin J treatment, according to our RNA-seq data. Importantly, clinical data revealed that ADAM9 expression are higher in PCa patients than in healthy controls and are associated with distant metastasis. Transfection with ADAM9 cDNA reverses Ugonin J-regulated downregulation of EMT, migration, and invasion in PCa cells. Ugonin J inhibits ADAM9-dependent motility by downregulating the phosphoinositide 3-kinase (PI3K), protein kinase B (Akt) and nuclear factor-κB (NF-κB) pathways.

**Conclusions:**

Our evidence suggests that Ugonin J is a novel therapeutic candidate for further development as a treatment for metastatic PCa.

## 1 Introduction

Prostate cancer (PCa) is a widely occurring cancer and a significant worldwide origin of male mortality [1]. Estimates suggest that in 2025, more than 300,000 men in the US will be diagnosed with PCa, and over 35,000 will die from it [2]. Due to its initial lack of symptoms, detecting PCa can be difficult, highlighting the need for vigilance regarding early indicators. PCa encompasses a wide spectrum of disease types, from those that develop slowly to those that are highly aggressive and fatal [3]. The main reason for deaths related to PCa is metastatic disease. While localized PCa generally has a favorable treatment outcome, metastatic PCa remains incurable [4]. Most men with advanced PCa present with multiple metastases. Bone is the most general organ of distant metastasis in PCa, with approximately 70% of patients with late-stage disease exhibiting bone metastases at diagnosis [5].

Epithelial-mesenchymal transition (EMT) is a key pathway through which malignant epithelial cells develop a mesenchymal phenotype, gaining invasive and metastatic properties [6]. During carcinogenesis, these cells transition into highly invasive mesenchymal-like cells. Malignant epithelial cells progressively lose adhesion and tight junction factors, including E-cadherin and fibronectin, while upregulating transcription activators, including Snail and Slug, and mesenchymal factors, for instance, vimentin and N-cadherin, promoting a migratory and invasive ability [7]. Among EMT transcription factors, Snail and Slug are critical drivers of EMT in PCa tissues [8,9]. Previous studies have demonstrated that EMT-related transcription factors contribute to therapeutic resistance, stemness, and tumor recurrence in PCa [10]. However, therapeutically targeting these transcription factors remains a challenge in developing effective EMT inhibitors.

A disintegrin and metalloproteinase domain-containing protein 9 (ADAM9) is part of the ADAM family of transmembrane proteins and belongs to the zinc protease superfamily. ADAM9 controls cell migration and molecular signaling in various malignancies [11–13]. In PCa, ADAM9 is a well-characterized protein with elevated levels in malignant prostate tissue, significantly correlating with shorter disease-free recurrence times [14,15]. ADAM9 expression is associated with PCa malignancy and recurrence [16,17]. Knockdown of ADAM9 inhibits PCa cell proliferation and invasion, processes related to cancer progression and metastasis [16]. These results indicated that ADAM9 is a critical target for treating PCa progression and metastasis.

Synthetic molecules and natural compounds based on natural models have received considerable attention because of their low toxicity and biological functions [18,19]. Heterocyclic compounds have been reported to exhibit anticancer activity [20]. Ugonin-derived compounds, a type of prenylated flavonoids extracted from the medicinal plant *Helminthostachys zeylanica*, have attracted scientists’ attention due to their pharmacological features [21]. Emergency reports have indicated that Ugonins exhibits various bioactivities, including anti-inflammatory, hepatoprotective, and anti-osteoporosis functions, and even anti-metastatic properties [22,23], which enhanced the anti-neoplastic potential of this medicinal plant. However, the role of this type of compounds in PCa remains largely unknown. This study examined the impact of Ugonin J on PCa metastasis and delved into the specifics of its mechanism.

## 2 Materials and Methods

### 2.1 Materials

**Figure 1:**
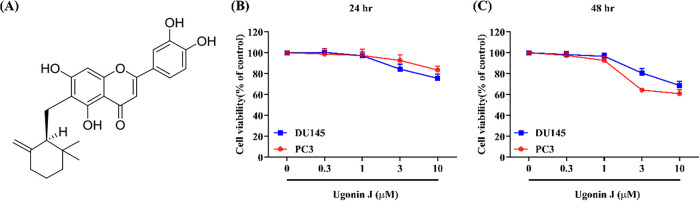
Effect of Ugonin J on the viability of PCa cells. (**A**) Chemical structure of Ugonin J. (**B**,**C**) Ugonin J does not affect the cell viability of PCa cells. PC3 and DU145 cells were treated with various concentrations of Ugonin J for 24 or 48 h, and cell viability was measured using the MTT assay. (n = 3)

Ugonin J was synthesized by Dr. Chih-Chuang Liaw (National Sun Yat-sen University, Taiwan), utilizing the process described in an earlier report (Fig. 1A) [24,25]. The purity of Ugonin J was >95%. The ADAM9 (SC-135822), Akt (SC-5298), β-actin (SC-47778), β-Catenin (SC-133240), p-p85a (SC-12929), p85 (SC-1637), p65 (SC-8008), Snail (SC-271977), and Slug (SC-166476) antibodies, PI3K activator (PI3K activator; SC-3036), AKT activator (Fumonisin B1; SC-201395), p65 activator (Prostratin; SC-203422) as well as ADAM9 siRNA (SC-41408) were purchased from Santa Cruz Biotechnology (Santa Cruz, CA, USA). E-cadherin (ab40772) and N-cadherin (ab76057) antibodies were bought from Abcam (Cambridge, MA, USA). p-Akt (4060S) and p-p65 (3033) antibodies were purchased from Cell Signaling Technology (Danvers, MA, USA). ADAM9 cDNA plasmid was obtained from Origene Technologies (RC222453; Rockville, MD, USA).

### 2.2 Cell Culture

The human prostate cancer cell lines PC3 (CRL-1435) and DU145 (HTB-81) were acquired by the American Type Culture Collection (Manassas, VA, USA). Cells were grown at 37°C in a humidified atmosphere containing 5% CO_2_, using RPMI-1640 media (Gibco, Rockville, MD, USA) supplemented with 10% FBS. All cell lines have been authenticated using short tandem repeats (STR). Contamination by Mycoplasma was regularly examined using PCR analysis. Ugonin J was dissolved in dimethyl sulfoxide (DMSO) to prepare a 10 mM stock solution and diluted in culture medium to the indicated concentrations for treatment.

### 2.3 MTT Assay

An MTT assay was used to examine cell viability, in accordance with our earlier publications [26]. 100 μL of culture media was used to seed PC3 and DU145 cells (5 × 10^3^) onto 96-well plates. Following a 24-h incubation period to promote cell adhesion, cells were incubated to varying doses of Ugonin J (0–10 μM) for a 24-h period before undergoing a cell-viability test. MultiskanTM FC microplate reader (Thermo Fisher Scientific, Waltham, MA, USA) will be used to measure the absorbance at 570 nm with the MTT reagent (M2128; Sigma-Aldrich, St. Louis, MO, USA).

### 2.4 RNA Sequencing (RNA-Seq) and Data Analysis

As per the manufacturer’s instructions, total RNA was extracted from PC3 cells treated with or without Ugonin J (3 μM) using TRIzol reagent (2001; Cyrusbioscience, Taipei, Taiwan). The isolated RNA samples were utilized for the subsequent library preparation. Then, libraries with different indices were multiplexed and sequenced on an Illumina HiSeq/Illumina Novaseq/MGI2000 instrument (Illumina, San Diego, CA, USA) using a 2 × 150 paired-end configuration as per the manufacturer’s guidelines. Heatmaps and volcano plots were generated to analyze differentially expressed genes using the ‘DESeq2’ (v1.26.0) in R (v3.6.3) within the Bioconductor (v3.10) package. We utilized a |log2fold change (FC)| > 1 with adjusted *p* value < 0.05 as the threshold for significantly differential expression [27].

Differentially expressed genes (DEGs) identified from RNA-seq analysis were submitted to the Gene Ontology (GO) and Kyoto Encyclopedia of Genes and Genomes (KEGG; https://www.genome.jp/kegg/pathway.html, accessed on 01 October 2025) database to explore potential pathways and analyze biological functions.

### 2.5 Bioinformatic Analysis

ADAM9 levels in normal prostate tissue, primary PCa, and lymph node metastatic tissues were analyzed using the University of Alabama at Birmingham CANcer data analysis Portal (UALCAN: https://ualcan.path.uab.edu/?utm_source=chatgpt.com, accessed on 01 October 2025) dataset. Additionally, ADAM9 levels in normal, primary, and metastatic PCa tissues, stratified by cancer stage, were evaluated using the TNMplot (https://tnmplot.com/?utm_source=chatgpt.com, accessed on 01 October 2025) dataset. Gene Expression Profiling Interactive Analysis (GEPIA: https://gepia.cancer-pku.cn/?utm_source=chatgpt.com, accessed on 01 October 2025) was performed to assess ADAM9, Snail, and Slug expression levels in healthy prostate and tumor tissue samples from The Cancer Genome Atlas (TCGA).

### 2.6 Reverse Transcription-Quantitative PCR (RT-qPCR) Analysis

PC3 and DU145 cells (~1 × 10^4^) were plated in 6-well dishes and incubated with Ugonin J (0, 0.3, 1, or 3 μM) for 24 h at 37°C. Using the TRIzol reagent as per the manufacturer’s guidelines, a total of 1 μg of RNA was extracted from PC and DU145 cells. An oligo-DT primer was used to convert the RNA into cDNA. The SYBR Green (A46012; Thermo Fisher Scientific, Waltham, MA, USA) was utilized to combine a 100 ng cDNA sample with specific primers, using GAPDH as the internal control. The sequences of the PCR primer (5^′^-3^′^) were as follows: ADAM9 forward, CTTGC TGCGA AGGAA GTACCTG and reverse, CACTC ACTGG TTTTT CCTCGGC. Thermocycling conditions were as follows: Initial denaturation at 95°C for 10 min, followed by 40 cycles of 95°C for 15 s and 60°C for 1 min. A StepOnePlus sequence detection system (v2.4; 4444202; Thermo Fisher Scientific, Waltham, MA, USA) was used to perform the qPCR tests in triplicate. The expression of ADAM9 mRNA was assessed via the ∆Cq comparative methods. To calculate relative expression, the comparative CT approach was employed [27,28].

### 2.7 Western Blot Analysis

RIPA lysis buffer (P0013; Beyotime Institute of Biotechnology, Shanghai, China) was used to extract total protein from PC3 and DU145 cells. In each lane, a total of 30 μg of protein was loaded, as calculated using the BCA kit (23225; Thermo Fisher Scientific, Waltham, MA, USA). Proteins were separated with resolving gels of 8% or 10%. After being separated through SDS-PAGE, proteins were transferred to ${\mathrm{Immobilon}}^{®}$ PVDF membranes. A 5% non-fat milk solution was used to block the membrane for 1 h at room temperature. The membranes were incubated with the primary antibodies overnight at 4°C, followed by a horseradish peroxidase-conjugated secondary antibodies (goat anti-rabbit IgG, SC-2357; 1:3000; goat anti-mouse IgG, SC-516102; 1:3000; Santa Cruz Biotechnology; Santa Cruz, CA, USA) for 1 h at room temperature. All antibodies were used at a final dilution of 1:1000. The expression of the target protein was measured using an ECL kit (WBULS0100; Millipore Sigma, St. Louis, MO, USA) and visualized with an ImageQuantTM LAS 4000 biomolecular imager (GE Healthcare, Little Chalfont, UK) [29,30].

### 2.8 Prostate Cancer Cells Migration and Invasion Assay

Migration experiments were conducted using Transwell inserts (Costar, Corning Incorporated, New York, NY, USA; 8-mm pore size) in 24-well plates. Prior to the migration and invasion assay, PC3 and DU145 cells were pretreated with Ugonin J (0, 0.3, 1 or 3 μM) for 30 min. In the upper compartment, there were approximately 1 × 10^4^ cells. For an invasion experiment, the same protocol was followed, but instead, 5 × 10^4^ cells were applied to a top chamber coated with Matrigel (BD Biosciences, Bedford, MA, USA). The cells were preserved using 3.7% formaldehyde for five minutes, incubated at 37°C with 5% CO_2_ for 24 h, and stained with PBS containing 0.05% crystal violet. Using cotton-tipped swabs, cells were taken off the upper side of the filters, which were then washed with PBS (0.01 M, pH 7.4) [31–33].

### 2.9 Cell Treatment and Transfection

After seeding the PC3 and DU145 cells (5 × 10^5^ cells/well) onto 6-well plates, the cells were pre-treated with PI3K (10 μM), Akt (10 μM) or NF-κB (10 μM) activators for 30 min or transfected with ADAM9 cDNA plasmid (1 μg) or ADAM9 siRNA (10 μM) for 24 h, using Lipofectamine 2000 transfection reagent (11668019; Thermo Fisher Scientific, Waltham, MA, USA) in accordance with the manufacturer’s instructions, then treated with Ugonin J (3 μM) for 24 h.

### 2.10 NF-κB Luciferase Assays

After transfection of the cells with an NF-κB firefly luciferase plasmid (Stratagene; St. Louis, MO, USA) using Lipofectamine 2000 (11668019; Thermo Fisher Scientific, Waltham, MA, USA), Ugonin J was stimulated for 24 h. Luciferase activity was measured using the dual luciferase assay system (E1910, Promega; Madison, WI, USA) following the manufacturer’s instructions. To account for transfection efficiency, Firefly luciferase activity was normalized against Renilla luciferase activity. The resulting data were presented as relative luciferase activity in comparison to untreated control cells [34,35].

### 2.11 Statistical Analysis

Statistical analyses were performed using GraphPad Prism 8.2 (GraphPad Software, San Diego, CA, USA). Data are presented as the mean ± standard deviation (SD) from at least three independent biological replicates. Statistical comparisons among more than two groups were analyzed by one-way ANOVA with Bonferroni’s post hoc test. Differences between groups were considered significant if the *p*-value was < 0.05.

## 3 Results

### 3.1 Ugonin J Blocks EMT, Migration and Invasion in PCa

**Figure 2:**
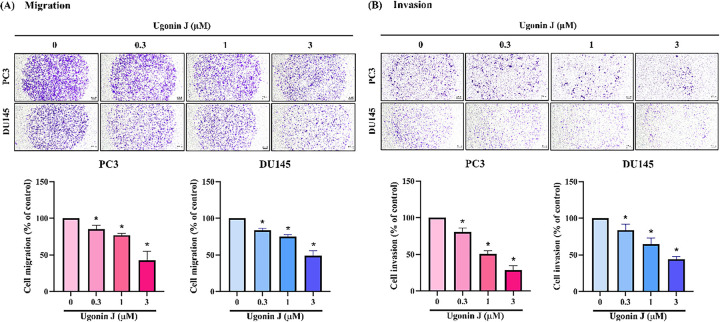
Ugonin J inhibits PCa cells’ migration and invasion activity. (**A**,**B**) Cells were treated with various concentrations of Ugonin J for 24 h, and a cell migration and invasion assay was performed using by Transwell assay. (n = 3). Scale bar = 200 μm. *p < 0.05 compared with the control group

**Figure 3:**
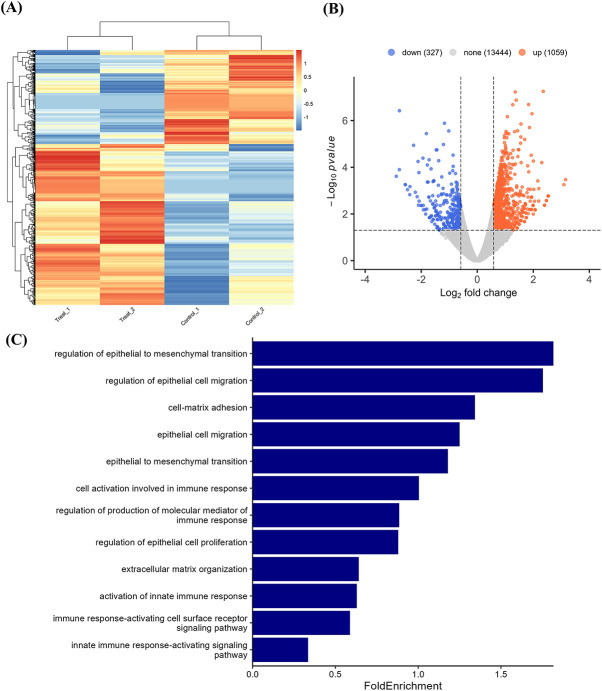

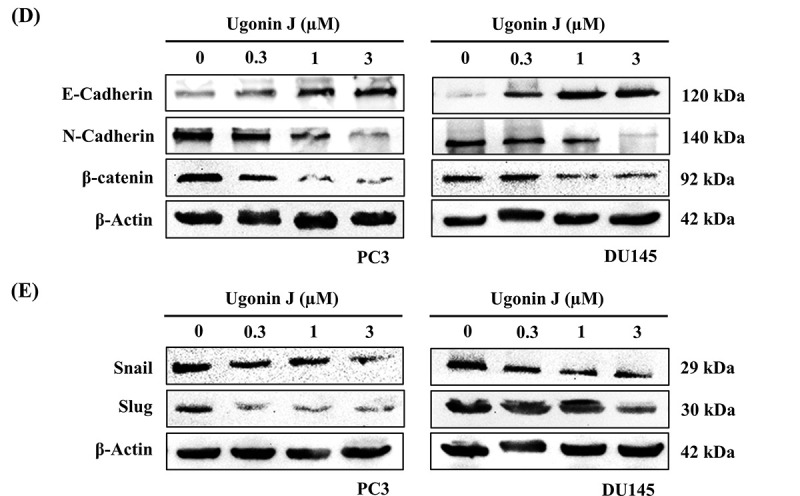
Ugonin J regulates EMT properties in PCa cells. (**A**) The result of a heatmap of RNA sequencing showing differentially expressed genes in PC3 cells with or without Ugonin J treatment. (**B**) The volcano plot shows the fold change in gene expression after Ugonin J treatment. (**C**) The biological processes and cellular functions are analyzed by the GO database. (**D**,**E**) Ugonin J regulates EMT protein expression in PCa cells. Cells were treated with Ugonin J for 24 h, and the protein expression of EMT markers was examined by Western blotting. (n = 3)

To investigate the roles of Ugonin J on migration and invasion, we first assessed its cytotoxicity in PCa cells. Ugonin J (0–10 μM) exhibited no significant cytotoxicity in PC3 and DU145 cells (Fig. 1B,C). We then evaluated Ugonin J’s inhibitory abilities on PCa cell migration and invasion after 24 h of treatment. Ugonin J significantly suppressed migration and invasion in PC3 and DU145 cells concentration-dependently (Fig. 2A,B). To investigate the molecular mechanisms behind Ugonin J’s anti-motility effects, we conducted RNA-seq analysis on PC3 cells treated with or without Ugonin J. Gene expression changes after Ugonin J treatment were revealed by the heatmap and volcano plot (Fig. 3A,B). GO biological process analysis indicated involvement in the regulation of epithelial to mesenchymal transition and cell motility, both linked to tumor metastasis (Fig. 3C). We therefore examined EMT markers after Ugonin J stimulation. The epithelial marker E-cadherin protein expression augmented after Ugonin J stimulation, but the mesenchymal indicators N-cadherin and β-catenin showed diminished expression (Fig. 3D). The transcriptional factors Snail and Slug are major activators of EMT [36]. Ugonin J treatment markedly diminished protein levels of Snail and Slug in PCa cells (Fig. 3E). These findings demonstrate that Ugonin J inhibits EMT, migration, and invasion in PCa cells.

### 3.2 ADAM9 Mediates Ugonin J-Augmented Inhibition of PCa Cell Migration and Invasion

**Figure 4:**
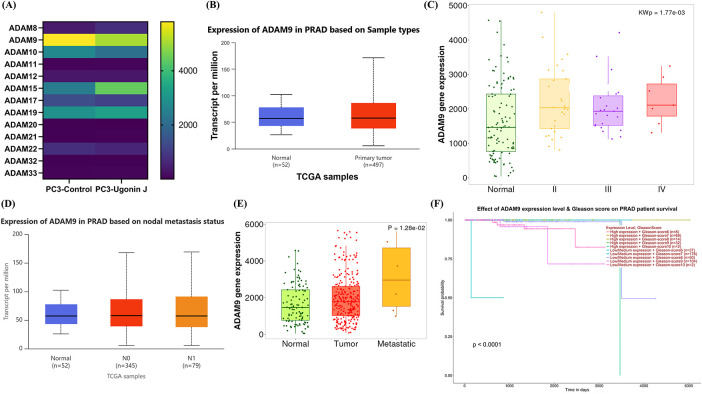
ADAM9 is associated with the progression and metastasis of PCa in patients. (**A**) ADAMs expression levels in control compared with PC3 cells treated with Ugonin J were analyzed by RNA-seq. (**B**) ADAM9 mRNA levels in normal and primary PCa tissues from the UALCAN dataset (*p* = 0.0217). (**C**) Correlation between ADAM9 and PCa cancer stage from the TNMplot dataset (*p* = 0.0017). (**D**) ADAM9 mRNA levels in normal and lymph node metastatic tissues from the UALCAN dataset showed significant upregulation in metastatic tissues compared with normal tissues (normal vs. N0, *p* = 0.041; normal vs. N1, *p* = 0.0178). (**E**) ADAM9 mRNA levels in normal, primary, and metastatic PCa tissues from the TNMplot dataset (*p* = 0.0128). (**F**) Kaplan–Meier survival analysis of ADAM9 expression in PCa patients from the UALCAN database (*p* < 0.0001)

**Figure 5:**
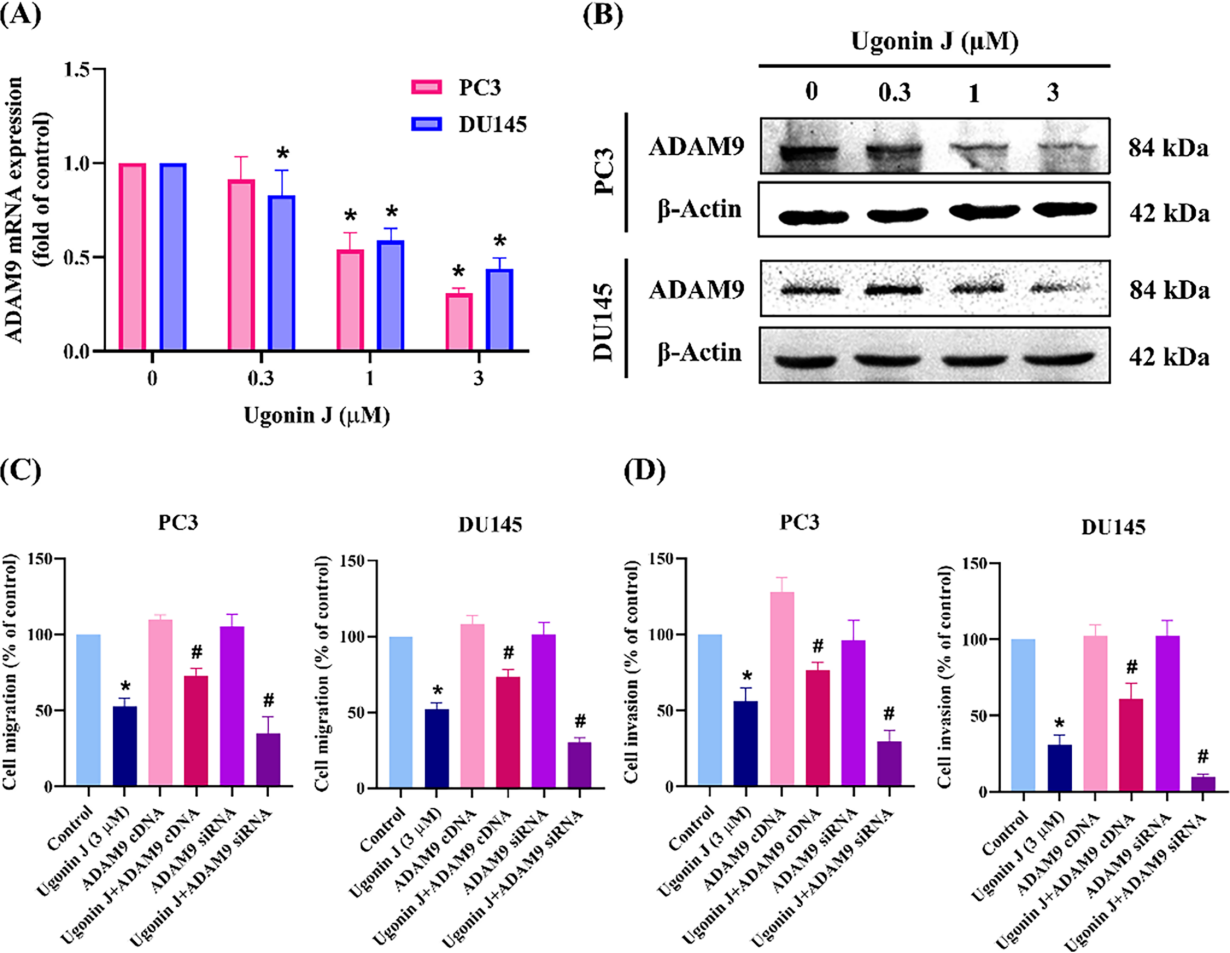

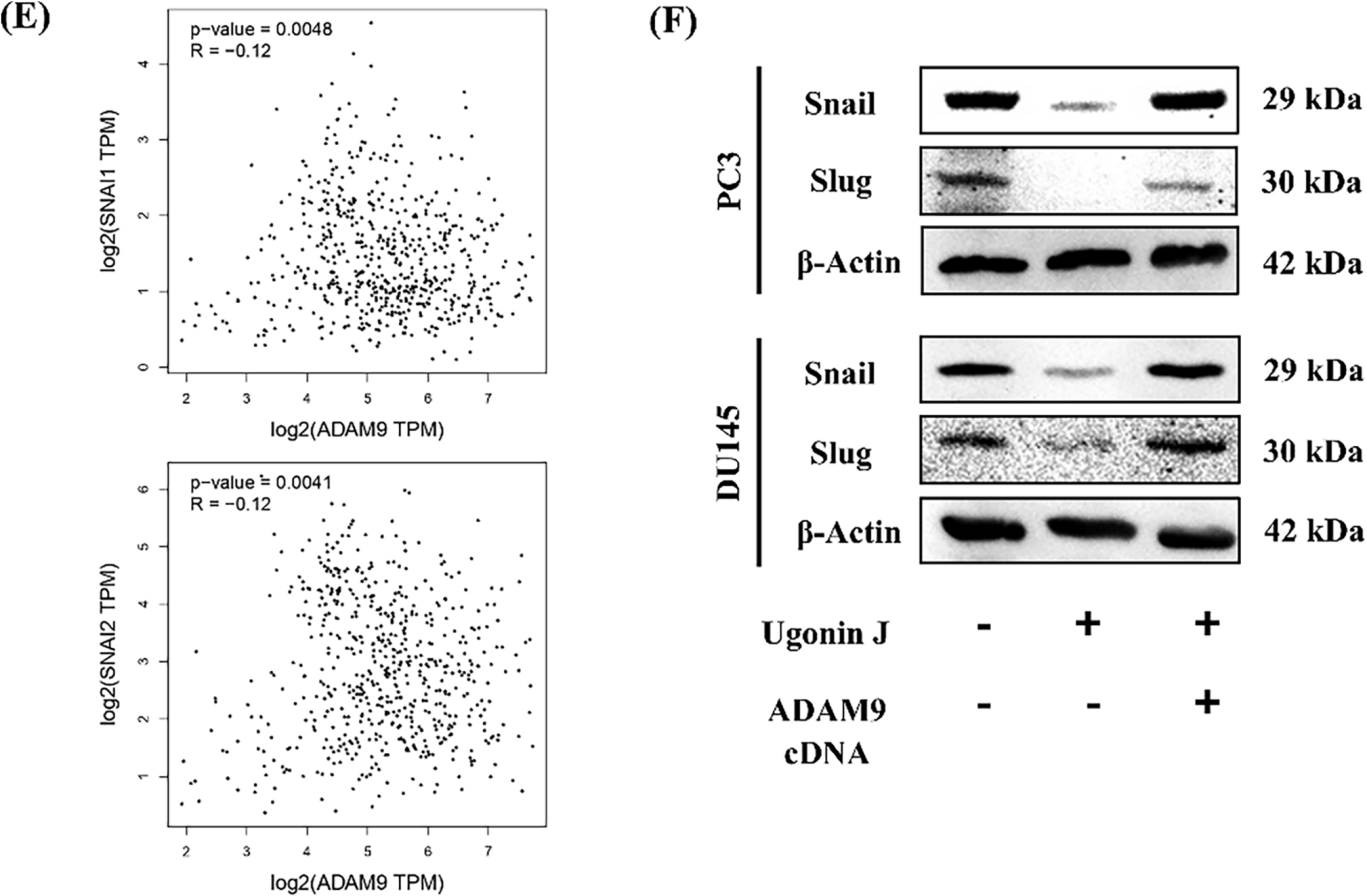
ADAM9 controls Ugonin J-induced inhibition of PCa cell migration and invasion. (**A**,**B**) Ugonin J inhibits ADAM9 expression. Cells were treated with various concentrations of Ugonin J for 24 h, the ADAM9 expression was examined by RT-qPCR and Western blotting. (**C**,**D**) ADAM9 controls Ugonin J-inhibited PCa motility. Cells were transfected with ADAM9 cDNA or siRNA then treated with Ugonin J for 24 h, the migration and invasion were examined. (**E**) The correlation of ADAM9 expression with Snail and Slug expression in PCa patients was analyzed using the GEPIA online database. (**F**) ADAM9 controls Ugonin J-inhibited Snail and Slug expression. Cells were transfected with ADAM9 cDNA, then treated with Ugonin J for 24 h, the Snail and Slug expression was examined by Western blotting. (n = 3). *p < 0.05 compared with control group; ^#^*p* < 0.05 compared with the Ugonin J-treated group

ADAM family proteins are critical modulators of cancer cell adhesion and metastasis [11–13]. Our RNA-seq analysis revealed that ADAM9 is the most downregulated of 13 ADAM proteins following Ugonin J treatment (Fig. 4A). TCGA analysis demonstrated markedly higher ADAM9 levels in PCa tissues compared to adjacent normal tissues (Fig. 4B). ADAM9 expression levels were also linked with tumor stage, lymph node metastasis, and distant metastasis (Fig. 4C–E). Furthermore, Kaplan–Meier survival analysis using the UALCAN database revealed that PCa patients with high ADAM9 expression and high Gleason scores exhibited poorer survival rates (*p* < 0.0001; Fig. 4F). We therefore investigated the role of Ugonin J on ADAM9 synthesis. Treatment of PCa cells with Ugonin J concentration-dependently inhibited ADAM9 mRNA and protein expression (Fig. 5A,B). Transfection of PCa cells with ADAM9 cDNA reversed Ugonin J-mediated inhibition of cell migration and invasion (Fig. 5C,D). In addition, ADAM9 siRNA enhanced Ugonin J-mediated inhibition of cell motility (Fig. 5C,D). ADAM9 expression levels were positively correlated with Snail and Slug expression levels in PCa patients without adjustment for confounders (Fig. 5E). Furthermore, ADAM9 cDNA transfection antagonized Ugonin J-mediated suppression of Snail and Slug expression (Fig. 5F). Thus, Ugonin J inhibits PCa EMT, migration, and invasion by suppressing ADAM9 expression.

### 3.3 Ugonin J Exerts Suppressive Effects via the PI3K, Akt, and NF-κB Signaling Pathways

**Figure 6:**
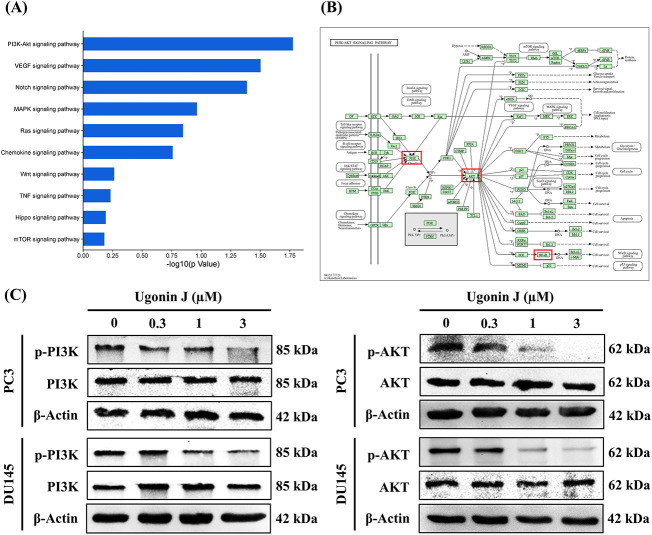

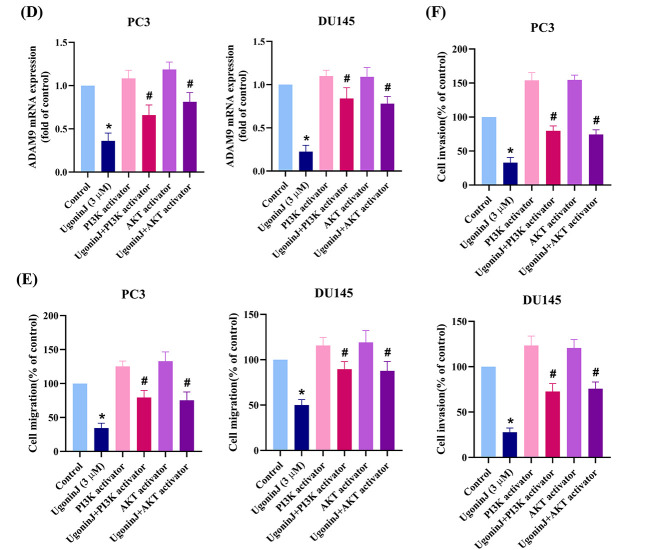
PI3K and Akt signaling pathways are involved in Ugonin J-regulated cell motility. (**A**) The cellular mechanisms are analyzed by the GO database. (**B**) Enrichment signaling pathways are analyzed by the KEGG database. (**C**) Ugonin J inhibits PI3K and Akt pathways. Cells were treated with Ugonin J for 24 h and the phosphorylation of PI3K and Akt was determined by Western blotting. (**D**–**F**) PI3K and Akt signaling pathways mediated Ugonin J-inhibited ADAM9 expression and cell motility. Cells were pre-treated with PI3K (10 μM) and Akt (10 μM) activators for 30 min, then treated with Ugonin J for 24 h, ADAM9 expression, cell migration and invasion assay was performed. (n = 3). *p < 0.05 compared with control group; ^#^*p* < 0.05 compared with the Ugonin J-treated group

**Figure 7:**
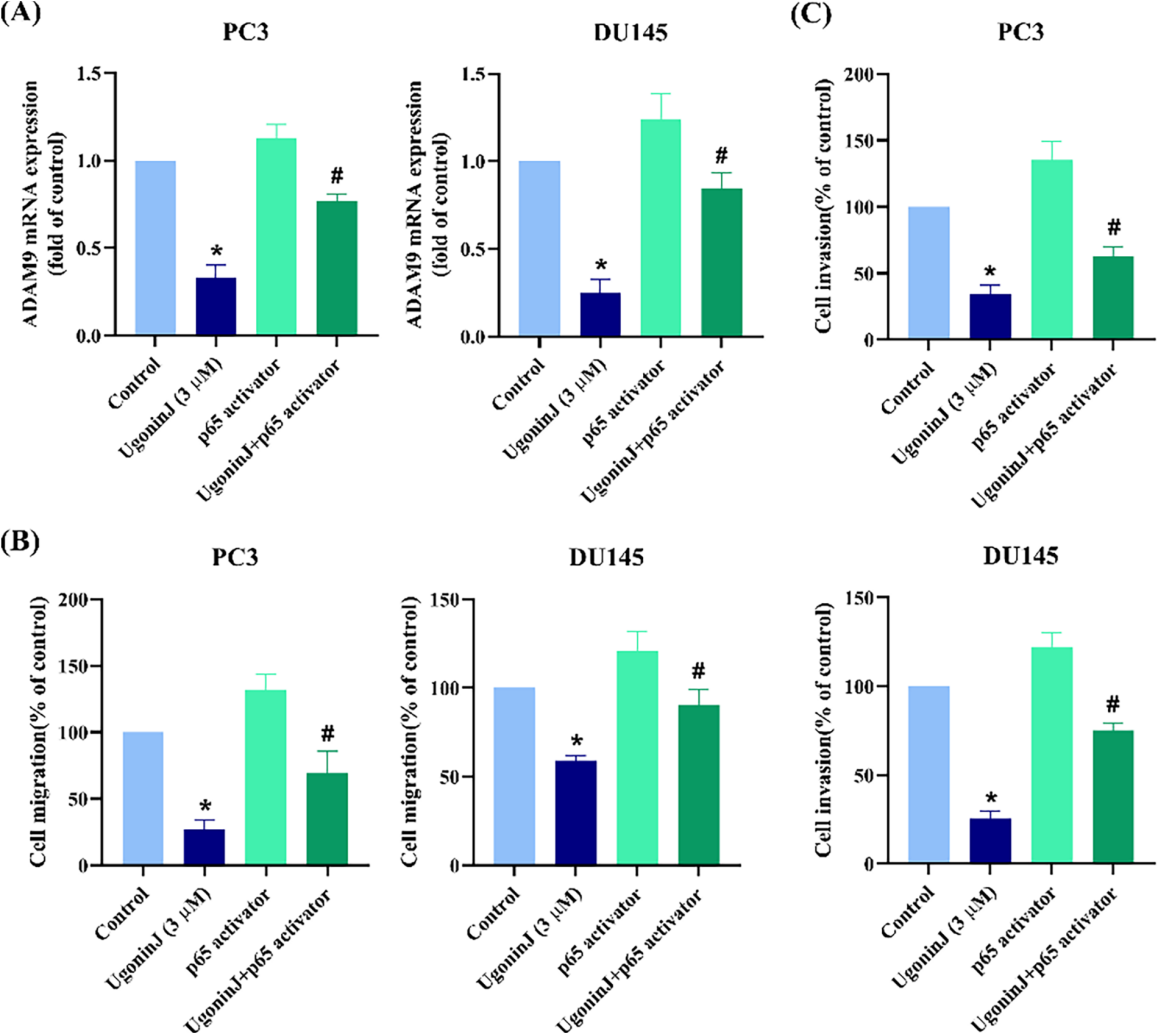

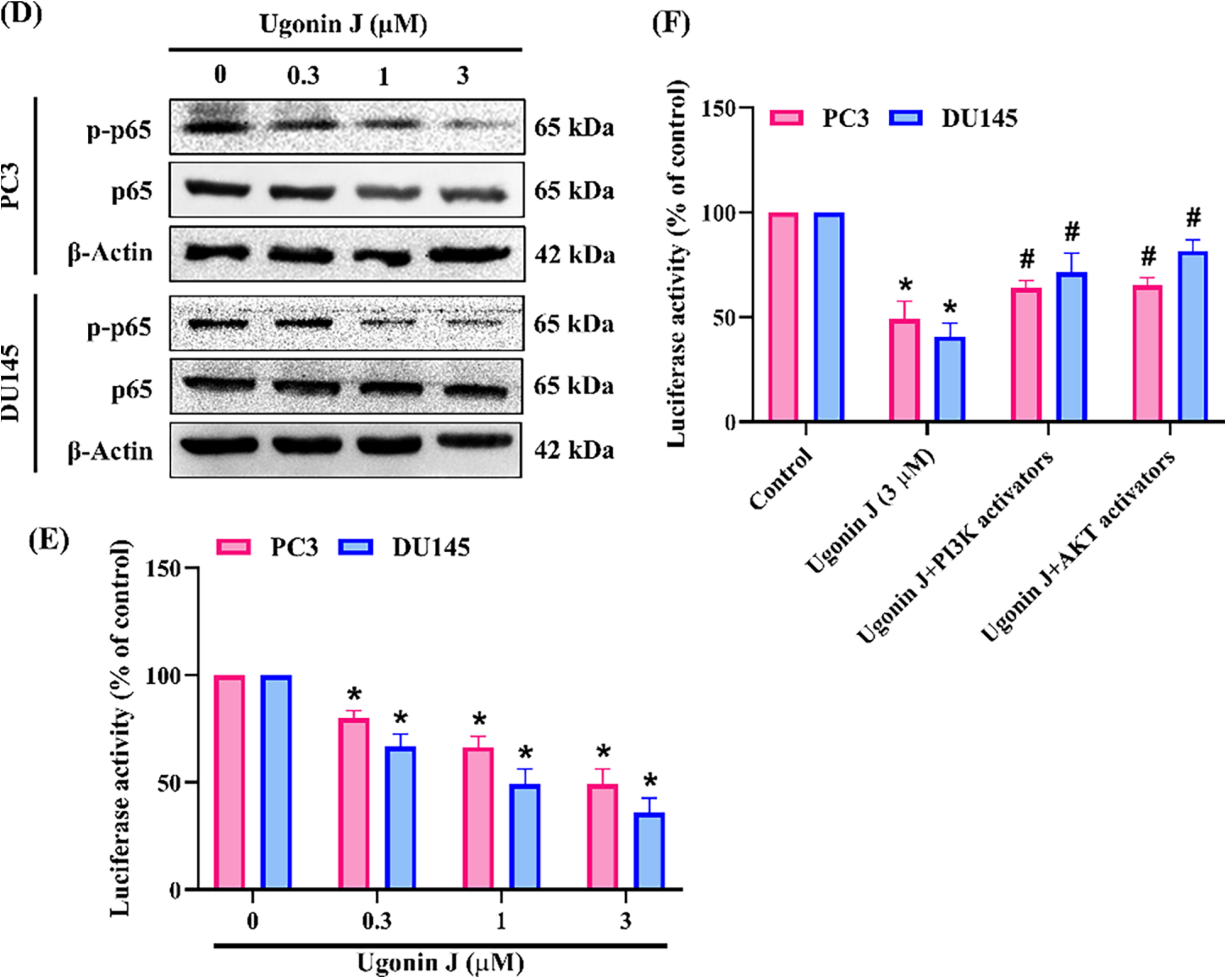
NF-κB pathway is involved in Ugonin J-mediated cell motility. (**A**–**C**) NF-κB mediated Ugonin J-inhibited ADAM9 expression and cell motility. Cells were pre-treated with NF-κB (10 μM) activator for 30 min, then treated with Ugonin J for 24 h, ADAM9 expression, cell migration and invasion assay was performed. (**D**) Ugonin J inhibits NF-κB pathway. Cells were treated with Ugonin J for 24 h and the phosphorylation of p65 was determined by Western blotting. (**E**,**F**) Cells were pre-treated with PI3K or Akt activators for 30 min, then treated with Ugonin J for 24 h, the NF-κB luciferase activity was performed. (n = 3). *p < 0.05 compared with control group; ^#^*p* < 0.05 compared with the Ugonin J-treated group

The PI3K-Akt signaling pathway, which encompasses the PI3K, Akt, and NF-κB mechanisms, was found to be mediated by Ugonin J through KEGG enrichment pathway analysis (Fig. 6A,B). Ugonin J treatment of PCa cells resulted in the inhibition of PI3K and Akt phosphorylation (Fig. 6C). The PI3K and Akt activators countered the reduction of ADAM9 production, migration, and invasion induced by Ugonin J (Fig. 6D–F). In addition, the use of an NF-κB activator during incubation counteracted the effects caused by Ugonin J (Fig. 7A–C). Ugonin J treatment of PCa cells inhibited p65 phosphorylation and NF-κB luciferase activity, as shown in Fig. 7D,E. Ugonin J also decreased the luciferase activity of NF-κB, which was reinstated through the activation of PI3K and Akt (Fig. 7F). Thus, Ugonin J mitigates ADAM9-mediated migration and invasion via the PI3K, Akt, and NF-κB pathways.

## 4 Discussion

PCa cases are diagnosed at a localized stage and generally have a favorable prognosis [37]. Metastatic and advanced PCa has limited therapy options, resulting to poor patient outcomes [38,39]. Researchers have been actively exploring more effective therapeutic strategies and medicines for PCa, such as targeted therapy, immunotherapy and hormone treatment [40]. In the last few years, several traditional Chinese herbal medicine components have demonstrated antitumor effects [41–43]. Ugonins, isolated from *H. zeylanica*, showed potent biological and pharmacological features, such as anti-inflammatory [25,44,45], immunomodulatory [46,47], and anti-cancer activities [22,23]. However, research on the anti-metastatic effects of Ugonin remains limited, with no prior studies investigating its impact on PCa metastasis. Here, we evaluated the effects of Ugonin J on PCa cell motility. Our findings demonstrate that Ugonin J inhibits PCa EMT, migration, and invasion by suppressing ADAM9 expression through the reduction of PI3K, Akt and NF-κB signaling pathways. We lacked sufficient quantities of other Ugonins or flavonoids to compare their effects on EMT and antimigratory activity in PCa cells with those of Ugonin J. The antimigratory activity of Ugonin J was observed at micromolar concentrations. Further studies are needed to use the Ugonin J structure as a lead compound to optimize its structure and evaluate its pharmacological effects and interacting proteins. The goal is to reduce the therapeutic concentration to nanomolar levels.

Multifunctional transmembrane proteases known as members of the ADAM family regulate cancer progression and motility by cleaving membrane-bound substrates, such as growth and cytokines mediators [48]. Numerous ADAMs show overexpression in cancers and are associated with unfavorable prognoses [49]. These proteases play a role in tumor growth by controlling cell migration, differentiation, immune evasion and apoptosis [49]. Among 13 ADAM proteins, ADAM9 is the most downregulated following Ugonin J treatment, according to our RNA-seq data. Ugonin J treatment also inhibits ADAM9 expression. Importantly, clinical data revealed that ADAM9 expression levels are higher in PCa patients than in healthy controls. Interestingly, transfection with ADAM9 cDNA reversed Ugonin J-mediated inhibition of PCa cell migration and invasion, indicating that ADAM9 is a critical modulator of Ugonin J-mediated suppression of PCa migration, invasion and metastasis.

The majority of PCa-associated deaths are caused by metastatic disease rather than the original tumor, and metastasis is a multi-step biological process. Notably, EMT plays a vital role in tumor progression and is key for cancer migration and metastasis. It is believed that cancer cells activate EMT, which helps them separate from the main tumor and enter the blood arteries [50]. Epithelial cells lose their apical-basal polarity and adherens junctions during EMT, acquiring a mesenchymal phenotype with increased motility. Therefore, EMT inhibition is an attractive therapeutic strategy. The data of this investigation showed that Ugonin J therapy in PCa prevents EMT in PCa cells by increasing the expression of epithelial cell markers and diminishing mesenchymal cell markers. EMT advancement during PCa metastasis is linked to transcription mediators of the Snail family, which includes Snail and Slug [50]. Our study indicated that Ugonin J inhibited Snail and Slug protein expression. Overexpression of ADAM9 cDNA abolished Ugonin J-controlled inhibition of Snail and Slug expression in PCa cells. Thus, Ugonin J reduces EMT and motility in PCa cells by suppressing ADAM9 expression. Our clinical dataset data indicate that high ADAM9 expression levels are associated with PCa progression, metastasis, and poor survival. Additionally, ADAM9 expression levels were positively correlated with the expression of EMT markers Snail and Slug in PCa patients. Thus, ADAM9 may serve as a potential biomarker for aggressive PCa and warrants comparison with other EMT-associated molecules. In the context of drug discovery, probing possible molecular mechanisms is vital. In this study, the PI3K-Akt signaling pathway, which includes components such as PI3K, Akt, and NF-κB, was identified as a major candidate in Ugonin J-treated PCa using RNA-seq analysis. The PI3K-Akt signaling cascades are essential to various cellular processes, such as, differentiation, inflammation, apoptosis, and migration [51,52]. Ugonin J reduces the activation of PI3K and Akt. The PI3K and Akt activators counteracted the Ugonin J-mediated inhibition of ADAM9 production, as well as cell migration and invasion. NF-κB is a crucial downstream target of the PI3K/Akt cascades, controlling tumor metastasis [53,54]. Notably, Ugonin J treatment suppressed the phosphorylation of p65. Moreover, the NF-κB activator counteracted the inhibitory effects of Ugonin J. PI3K, Akt, and NF-κB activators all reversed Ugonin J-mediated inhibition of ADAM9, indicating that ADAM9 is a downstream effector of these pathways. The restoration of NF-κB luciferase activity suppression by Ugonin J through PI3K and Akt activators suggests that the pathways of PI3K, Akt, and NF-κB are involved in regulating the anti-motility effects of Ugonin J in PCa cells. Here, we found that Ugonin J inhibited NF-κB promoter activity and ADAM9 expression. However, we lacked an ADAM9 promoter plasmid containing an NF-κB binding site to examine whether NF-κB directly binds to the ADAM9 promoter. Further investigation is needed to confirm the direct binding of NF-κB to the ADAM9 promoter.

There are multiple limitations to this study. First, the absence of cytotoxicity data in normal prostate epithelial cells restricts our ability to comprehensively evaluate the safety profile of Ugonin J in non-cancerous contexts. This will be addressed in forthcoming research, which will assess the impact of Ugonin J on normal prostate epithelial cell lines. Second, the current research provides strong mechanistic and cell-based evidence, but it is limited by the absence of *in vivo* validation because Ugonin J is not readily available for large-scale experiments. Further research should evaluate its effectiveness in *in vivo* metastatic PCa models. Third, we lack sufficient quantities of other Ugonins to compare their antimigratory effects in PCa cells with those of Ugonin J. Further studies are needed to determine the optimal Ugonin structure.

**Figure 8:**
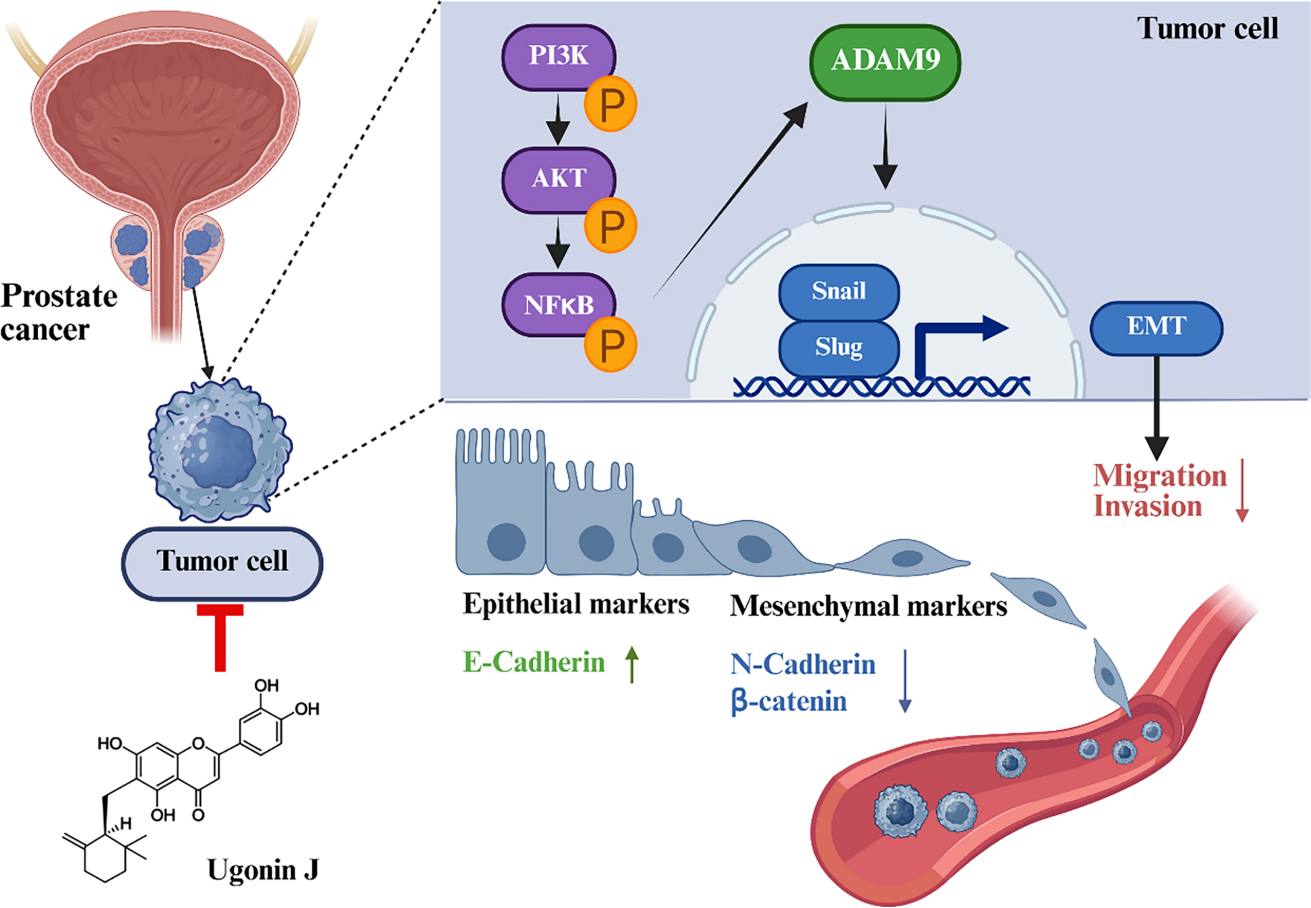
Schematic diagram summarizing the mechanism by which Ugonin J inhibits PCa migration and invasion. Ugonin J inhibits migration and invasion in PCa cells. Ugonin J restricts EMT, migration, and invasion by inhibiting the expression of ADAM9 through suppression of the PI3K, Akt and NF-κB pathways. (P) indicate phosphorylation; (↑) and (↓) indicate upregulation and downregulation, respectively. Illustration created with BioRender.com

In conclusion, this research showed for the first time that Ugonin J, a prenylated flavone from *Helminthostachys zeylanica*, blocks migration and invasion in PCa cells. Ugonin J restricts EMT, migration, and invasion by inhibiting the generation of ADAM9 through inhibition of the PI3K, Akt, and NF-κB pathways (Fig. 8). Our findings suggest that Ugonin J could be a novel therapeutic candidate for further development in treating metastatic PCa.

## Data Availability

The datasets used and/or analyzed during the current study are available from the corresponding authors on reasonable request.
